# Disease Biomarkers for Precision Medicine: Challenges and Future Opportunities

**DOI:** 10.1016/j.gpb.2017.04.001

**Published:** 2017-04-07

**Authors:** Edwin Wang, William C.S. Cho, S.C. Cesar Wong, Siqi Liu

**Affiliations:** 1 Center for Health Genomics and Informatics, University of Calgary Cumming School of Medicine, Calgary, AB T2N 4N1, Canada; 2 Department of Clinical Oncology, Queen Elizabeth Hospital, Hong Kong Special Administrative Region; 3 Department of Health Technology and Informatics, Faculty of Health and Social Sciences, Hong Kong Polytechnic University, Hong Kong Special Administrative Region; 4 CAS Key Laboratory of Genome Sciences and Information, Beijing Institute of Genomics, Chinese Academy of Sciences, Beijing 100101, China

Most of the human diseases are complex diseases, which could be caused by many genetic pathways. This means that for a given phenotype (*i.e.*, a complex disease), there are multiple potential genes which could be genomically or epigenetically changed (*i.e.*, mutations, copy number variations, epigenetic modifications, and so on). Therefore, it is understandable that different individuals who share the same phenotype/diseases may have different causal genes and thus, may have different drug targets. For example, mutated genes are rarely common between the cancer patients of the same cancer type [1]; furthermore, for a given drug, only 10%−30% of the patients of the same cancer type respond to that drug [2]. It has been suggested that genomic and other omic features and/or environmental and lifestyle factors, could contribute to these differences such as drug response. It is clear that we should give the ‘right drug’ to the ‘right patient’ at the ‘right time’. One of the missions of the many ongoing precision medicine programs is to reach this goal using omic (*i.e.*, genomic, proteomic, epigenetic, and so on) and/or environmental and lifestyle factors of the individuals.

High-throughput technologies drive the evolution of biology and medicine. To realize precision medicine, it is essential to identify biomarkers using either omic data alone or in combination with environmental/lifestyle factors. The challenge is how to transform the data into biomarkers that could predict clinical outcomes, drug response or others. In general, it is difficult to identify ‘high-quality’ biomarkers which have high accuracy and robustness [3], [4] using omic data such as gene expression data, proteomic data and so on. For example, many omic-based cancer biomarkers are not robust, meaning that a biomarker identified from a patient cohort loses its predictive power in other cohorts of the same cancer type/subtype [3], [5]. Efforts have been made to develop new algorithms to overcome this problem. For example, Multiple Survival Screening (MSS) and Significance Analysis of Prognostic Signatures (SAPS) have been developed for identifying robust cancer biomarkers [4], [6], [7].

In the post-genome sequencing era, genome sequencing gets cheaper and cheaper, which makes genome sequencing become affordable and accessible to the clinic. Therefore, it is very attractive to identify biomarkers using the whole-genome/whole-exome sequencing data. Nonetheless, given the aforementioned features of the complex diseases, it has proven challenging to construct predictive models (*i.e.*, identify biomarkers) using the whole-genome/whole-exome sequencing data [8]. Because multiple gene interactions govern the underlying molecular mechanisms of the complex diseases, the linear model approach is not an option for identifying biomarkers using the genome sequencing data. A network-based, non-linear approach could hold promise to solve this problem. For example, the Cancer Hallmark Network Framework (CHNF) [9] provides a solution to the problem. Recently, a CHNF-based algorithm has been developed to successfully predict breast cancer recurrence using the whole-exome sequencing data of the tumors (Milanese et al., unpublished data).

Omic profiling of cell-free DNA (*i.e.*, liquid biopsy) has opened a new avenue for identifying non-invasive biomarkers, which are extremely useful in clinics. Much more efforts will be made in this direction in the near future. In addition, the recent development of the single-cell omic technology could bring new opportunities for biomarker identification. Finally, almost all of the efforts made have focused on identifying biomarkers using omic data in the past. However, most of the diseases are caused by the interaction of genetics and environmental/lifestyle factors. Therefore, to accurately predict clinical features of the complex diseases, the future work should focus on identifying biomarkers by integrating the data of the omics and environmental/lifestyle factors.

## Competing interests

The authors have declared that there are no competing interests.
